# The mechanics of hyperactivation in adhered human sperm

**DOI:** 10.1098/rsos.140230

**Published:** 2014-10-01

**Authors:** E. H Ooi, D. J Smith, H Gadêlha, E. A Gaffney, J Kirkman-Brown

**Affiliations:** 1 School of Clinical and Experimental Medicine, University of Birmingham, Birmingham B15 2TT, UK; 2 School of Mathematics, University of Birmingham, Birmingham B15 2TT, UK; 3 Centre for Human Reproductive Science, Birmingham Women's NHS Foundation Trust, Birmingham B15 2TG, UK; 4 Wolfson Centre for Mathematical Biology, Mathematical Institute, University of Oxford, Oxford OX2 6GG, UK; 5 School of Engineering and Centre for Scientific Computing, University of Warwick, Coventry CV4 7AL, UK

**Keywords:** sperm motility, high-speed imaging, binding, 4-aminopyridine

## Abstract

Hyperactivation is an important phenomenon exhibited by mammalian sperm during the process of acquiring fertilization capacity. The majority of studies have focused on incubation-induced hyperactivation in non-human species, which typically differ in size, shape, and are more homogeneous than human sperm. We develop an alternative approach via drug-induction, using high-speed imaging and analysis of same-cell changes in the flagellar movement of adhered cells. Following stimulation with 4-aminopyridine, approximately two-thirds (21 of 34) of the cells analysed exhibited a waveform with a single characteristic frequency; in all cases, the frequency was lower than before stimulation. The remaining cells (13 of 34) exhibited a more complex motility with multiple-frequency modes. The lowest mode in all cases was lower than the frequency prior to stimulation. Flagellar bending increased in all cells following stimulation and was significantly greater in the multiple-frequency responders. Despite the increased bending, time-averaged hydrodynamic power dissipation decreased significantly when assessed across all cells, the effect being significantly greater in the multiple-frequency responders than single frequency. These results reveal the heterogeneity of responses of human sperm to a hyperactivating stimulus, the methodology being potentially useful for assessing dynamic responses to stimuli in human sperm, and physiological selection of cells for assisted reproduction.

## 2. Introduction

In human fertilization, an initial population of tens to hundreds of millions of sperm cells is reduced to only tens to hundreds near the site of fertilization, with usually at most a single-cell achieving fertilization. The processes underlying sperm migration, possible guidance and selection, and the final stages of binding to the egg and fertilization in the human are however only beginning to be understood. This study will focus on hyperactivation, a change in the beating behaviour of the sperm flagellum associated with the acquisition of fertilizing capacity. In particular, we will characterize kinematic features of the flagellar movement in adhered, rather than free-swimming, cells and also determine viscous power dissipation, that is, the rate at which mechanical work is performed by the flagellum on the surrounding medium owing to its movement.

Capacitation is a multifaceted series of structural and functional changes that must occur in mammalian sperm in the female reproductive tract in preparation for acrosome reaction and fertilization [1–3]. The associated motility change of hyperactivation was first documented in rodent sperm as highly vigorous beating occurring following incubation with follicular fluid [4–6] and subsequently studied in the human [7–10]. For further detailed review, see Mortimer *et al.* [11] for human sperm; while Chang & Suarez [12] consider the functional role of hyperactivation across mammalian species.

Hyperactivation is generally characterized as high amplitude flagellar bending, a reduction in beat frequency, and side-to-side yawing or non-progressive ‘star-spin’ motility in free-swimming cells in low viscosity fluids. Potential roles for this behaviour include enhanced migration through viscoelastic fluids lining the female reproductive tract and penetration of the layers surrounding the egg [13]. In adhered cells, imaging and mechanical calculations performed on monkey sperm have shown a large increase in tangential forces in the hyperactivated state [14]. Recent theoretical modelling [15] suggests that increased ‘tugging’ forces are exerted during hyperactivation, which may enhance detachment from epithelial binding; computer simulation has also been used to demonstrate that hyperactivation is important in producing the bind-and-release motility associated with sperm-tract interaction [16].

Experimentally, hyperactivated beating is generally induced by incubating cells in supplemented laboratory media; the proportion of cells performing high amplitude head movements increases with incubation time. Typically with this approach, the proportion of cells hyperactivating in a human sample is around or below 20% [8]. The incubation technique, combined with high-speed imaging, has enabled significant information to be extracted regarding human sperm flagellar characteristics during hyperactivation [11]; in the past decade, digital imaging methods have begun to provide detailed mechanistic characterization of non-human species [14,17]. An area which warrants further investigation is the effect of hyperactivation on power dissipation: calculations of this quantity show considerable variability in the literature for different species [18–20] and therefore it is of interest to define more precisely the changes in mechanical energy requirements and metabolic demands associated with human sperm hyperactivation.

In this study, we will examine same-cell changes in the flagellar beat of tethered sperm, stimulated to hyperactivate with a pharmacological agent and studied via digital imaging and mechanical analysis. This approach will enable hyperactivation to be studied at the level of a near-instantaneous dynamic change to the beating of individual flagella rather than as an averaged population-level change. The agent 4-aminopyridine (4AP) is a potent inducer of hyperactivation, which acts via elevation of intracellular calcium and action on calcium stores [21–23]. We will focus on detailed spatial–temporal measurement of the degree of bending, frequency domain characteristics of bending waves and power dissipation, before and after stimulus.

## 3. Material and methods

### 3.1 Donors and semen sample preparation

Four healthy donors with normal semen parameters were recruited at Birmingham Women's NHS Foundation Trust after giving informed consent. Semen samples were obtained through masturbation following 2–3 days' abstinence. Sperm were prepared by direct swim-up: aliquots of 300 μ*l* of the freshly collected raw semen were underlayered beneath 1 ml of Earle's Balanced Salt Solution (sEBSS) without phenol red, supplemented with 2.5 mM Na pyruvate and 19 mM Na lactate (06-2010-03-1B; Biological Industries, Kibbutz Beit HaEmek, Israel), and 0.3% *wt* *vol*^−1^ charcoal delipidated bovine serum albumin (Sigma; A7906) in Falcon 2007 tubes (Becton Dickinson, UK). The tubes were incubated at 37°C and 6% CO_2_ atmosphere for 60 min, following which motile sperm cells were collected by transferring the top 600 μ*l* of medium into a fresh tube.

### 3.2 Experimental procedures

A bespoke imaging chamber (Cairn Research Ltd) was used for imaging, consisting of two orifices (inlet and outlet) and two parallel coverslips (top and bottom). The inner surface of the top coverslip was coated with 0.1% poly-d-lysine (Becton Dickinson) and air-dried to enhance sperm-cell attachment to the surface of the coverslip. The chamber was filled with the motile sperm cells collected after incubation. A particular cell of interest adhered by its head onto the surface of the coverslip but with freely moving flagellum was identified and imaged for a duration of 5 s (1662 imaging frames) prior to stimulation with 4AP. The chamber was then manually perfused with 1 ml of medium containing 4AP at a concentration of 2.5 mM using a 5 ml syringe. Following stimulation, imaging was carried out for an additional 5 s. All experiments were performed with medium at room temperature (21°C).

### 3.3 Imaging and flagellar movement analysis

Cells were imaged using negative phase contrast microscopy (Olympus BX-50, objective 20×/0.50∞/0.17 Ph1). High-speed imaging was performed using a Hamamatsu Photonics C9300 CCD (pixel size: 7.4×7.4 μm, pixel sub-array 640×200, frame rate 332 Hz) without ‘binning’, streaming data to a Dell Precision 490 workstation, running Wasabi 1.5 software (Hamamatsu Photonics).

The flagellar capture and analysis were as described in [24]. One hundred continuous imaging frames (approx. 0.3 *s*) were sampled for analysis from the 1662 frames recorded; briefly, the flagellum was captured by thresholding and skeletonizing phase contrast images in Image-Pro Plus (Media Cybernetics, Rockville, MD, USA) and fitting cubic approximating splines in Matlab (Mathworks, Natick, MA, USA), producing a discretized representation ***x***(*s*_*j*_,*t*_*k*_), where *s*_*j*_ is arclength and *t*_*k*_ is timestep. Flagellar velocity and unit tangent were then calculated from centred differences as ***u***(*s*_*j*_,*t*_*k*_)≈(2*Δt*)^−1^(***x***(*s*_*j*_,*t*_*k*+1_)−***x***(*s*_*j*_,*t*_*k*−1_)) and ***T***(*s*_*j*_,*t*_*k*_)≈(2*Δs*)^−1^(***x***(*s*_*j*+1_,*t*_*k*_)−***x***(*s*_*j*−1_,*t*_*k*_)), respectively. The flagellum was discretized into 200 arclength points; resampling the cubic spline representation enabled the use of a finer temporal discretization of 400 time points from 100 imaging frames.

**Figure 1.**
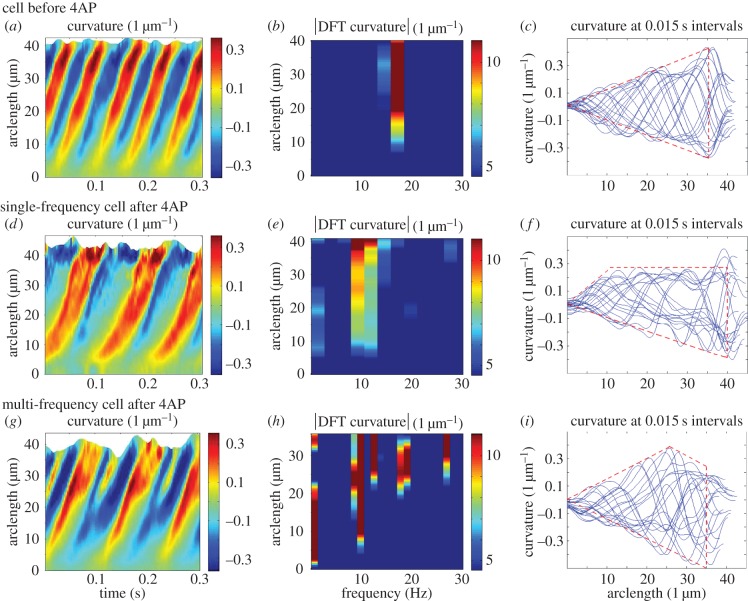
Plots of curvature of representative single- and multiple-frequency beat cells before and after stimulation with 4AP. Before stimulation with 4AP: (*a*) spatio-temporal distribution of curvature; (*b*) magnitude of the temporal discrete Fourier transform (DFT) of curvature as a function of frequency and arclength, which increases away from the head-flagellum junction; (*c*) curvature versus arclength at different time levels; and their corresponding plots after stimulation in (*d*–*f*), respectively, for the single-frequency beat cell and (*g*–*i*), respectively, for the multiple-frequency beat cell. Red dashes emphasize the variability in beat envelope between different cells.

**Figure 2.**
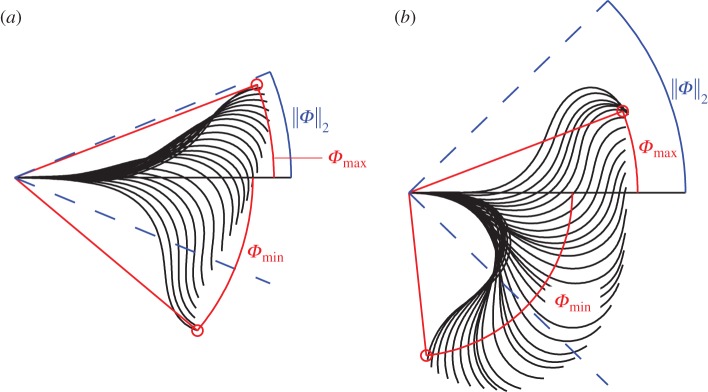
Definition of *proxidistal angle* *Φ* from proximal tangent to the point 30 μm from the head for the same cell (*a*) before 4AP and (*b*) after 4AP. The flagellar waveform is plotted relative to a set of axes fixed in the head; the figure shows only the portion of the waveform up to 30 μm arclength for clarity. The maximum and minimum values of $\Phi_{\min}$ and $\Phi_{\max}$ are marked, along with the RMS ∥*Φ*∥_2_.

Beat frequency was calculated from the period required to complete one beat cycle, assessed through curvature plots (e.g. figure 1); in addition, the absolute values of the temporal discrete Fourier transform of curvature were calculated following stimulation. To aid with the description of flagellar bending, we introduced the *proxidistal angle* *Φ*(*t*), defined as the maximum angle formed by a point at arclength distance 30 μm from the first point captured on the flagellum, measured relative to the tangent to the proximal midpiece. Figure 2 shows this quantity in the same cell, before and after hyperactivation. We report the temporal root mean squared (RMS) value $∥\Phi {∥}_{2}=(1/T){\left(\int_{0}^{T}\Phi {\left(t\right)}^{2}\;dt\right)}^{1/2}$, depicted by the blue wedge. The selection of a point 30 μm from the head obviates the difficulty with accurate resolution of the far-distal flagellum.

Hydrodynamic forces exerted by the sperm flagellum were estimated from the modified resistive force theory of Lighthill [25], with hydrodynamic force per unit length being calculated as

3.1 $$f(s,t)=K_{T}(u(s,t)\cdot T(s,t))T(s,t)+K_{N}(u(s,t)\cdot N(s,t))N(s,t),$$

where ***T*** and ***N*** denote unit tangent and normal vectors to the flagellum, and *K*_*T*_, *K*_*N*_ are tangential and normal resistance coefficients. Lighthill's ‘sub-optimal’ choice of

3.2 $$K_{T}=\frac{2\pi \mu }{\log(2q/a)}\;\mathrm{and}\;K_{N}=\frac{4\pi \mu }{\log(2q/a)+1/2}$$

have previously been shown to yield satisfactory accuracy [26]. The parameter *μ* denotes dynamic viscosity, taken as being equal to that of water at room temperature, 0.001 Pa s, the length scale *q*=0.09*Λ* based on Lighthill's analysis [25], with typical wavelength *Λ*≈40 μm measured from experimental data, and radius *a*≈0.5 μm based on the typical radius of the human sperm flagellum. Excessive precision in the choice of the values for *q*, *a* and *Λ* is not warranted, given the logarithmic dependence on these terms and the approximate nature of the hydrodynamic model.

Time-averaged power dissipation owing to the movement of the flagellum was calculated using the following formula:

3.3 $$\bar{P}=\frac{1}{T}\int_{0}^{T}\int_{0}^{L}f(s,t)\cdot u(s,t)\;ds\;dt.$$

The quantity *s* is an arclength parametrization of the flagellum measured from proximal to distal; *L* is the observed flagellar length and *T*=0.301 *s*, the period analysed. The relative contribution of power dissipation by head rotation about the tethering point is *O*(*α*^3^/(*A*^2^*L*)), where *α* is hydrodynamic radius of the head and *A* is beat amplitude. Because α≪A≲L, this quantity may be safely neglected.

### 3.4 Statistical analysis

Owing to the relatively small sample sizes and the fact that data were not clearly normally distributed, robust non-parametric tests were used. These tests remove the need for the underlying distributions to be normal, at the expense of some loss of power in detecting significant differences. The two-tailed Wilcoxon signed-rank test was used to assess the within-cell effect of 4AP stimulation on beat frequency, proxidistal angle and power. The two-tailed Mann–Whitney–Wilcoxon test was used to assess the percentage change in magnitude of proxidistal angle and power dissipation change between the single- and multiple-frequency subpopulations. Summary statistics were reported as median and interquartile range. Statistical calculations were carried out with the software package R [27].

## 4. Results

### 4.1 Dummy perfusion

Following perfusion with control (4AP-free) medium, five of 10 cells showed no detectable change in beat frequency (less than 1%), two cells showed a reduction in beat frequency of 5.9 and 8.1%, while two other cells showed an increase in beat frequency of 10.9 and 11.9%. The remaining cell showed between-beat heterogeneity resembling the multiple-frequency beat pattern described below, exhibiting more than one frequency peak in the power spectrum of the curvature.

### 4.2 Effects of 4-aminopyridine on flagellar kinematics

**Figure 3.**
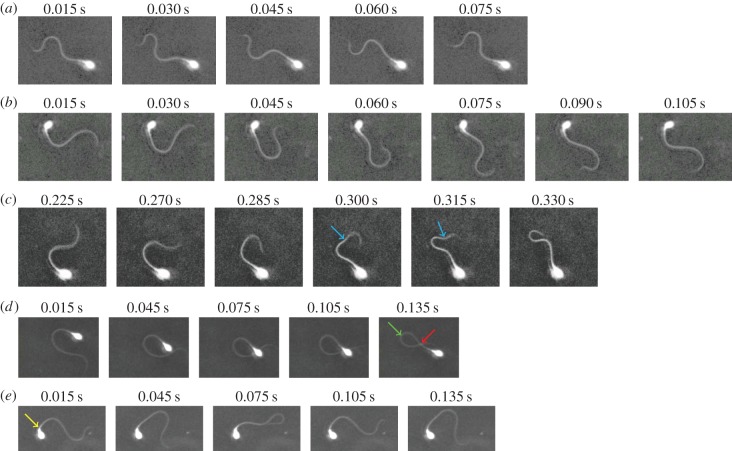
Imaging frames of representative cells (*a*) before 4AP stimulation and (*b*–*e*) after stimulation, exhibiting (*b*) single-frequency and (*c*) multiple-frequency beating (cyan arrow indicates an apparent secondary wave). Two cells were excluded from analysis owing to overlap of the flagellum (indicated by red arrow); these cells exhibited: (*d*) severe bending of the flagellum (green arrow) and (*e*) midpiece kink (yellow arrow).

We now report observations of cells before and after perfusion with 4AP. Images of nine different cells were recorded for each of the four donors. Prior to 4AP stimulation, the cells exhibited consistent beat patterns; characteristics which include almost symmetrical flagellar waves, very little bending near the proximal midpiece and minimal rotational movement around the pivoting point (figure 3*a*). The propagation of waves can be visualized in the form of a curvature plot (figure 1*a*) with diagonal blue and red streaks indicating waves of bending in opposite directions; plotting the magnitude of the signal in the frequency domain (figure 1*b*) reveals a dominant beat frequency which is similar throughout the flagellar length. The degree of bending, visualized via a timelapse plot of curvature, generally increases throughout most of the flagellar length, as reported previously ([24] and red dashed lines in figure 1*c*).

Of the 36 cells imaged, only 34 were successfully analysed both before and after stimulation; the remaining two exhibited flagellar movement significantly out of the plane of focus (figure 3*d*,*e*), which prevented their analysis. The cell shown in figure 3*e* showed a severe bend or kink in the proximal midpiece following stimulation, while the rest of the flagellum continued to propagate bending waves (figure 3*e*). When the imaging chamber was further perfused with sEBSS to remove the 4AP stimulant, the kink disappeared and the cell returned to its normal morphology.

### 4.3 A subpopulation of cells respond with multiple frequency peaks; primary beat frequency is consistently reduced by 4-aminopyridine

The very lowest, essentially zero frequency, in figure 1*e*,*h* simply reflects the time-averaged asymmetry of the flagellar waveform rather than the timescales associated with the flagellar beat and are not considered further in the discussion of frequency. With this qualification, we have that following stimulation, 21 cells exhibited single-frequency flagellar beats (figures 1*d*–*f* and 3*b*), whereas the remaining 13 cells exhibited multiple-frequency flagellar beats (figures 1*g*–*i* and 3*c*). For the latter cells, we analysed the two lowest non-zero frequencies, identified as ‘mode I’ (primary) and ‘mode II’ below.

**Figure 4.**
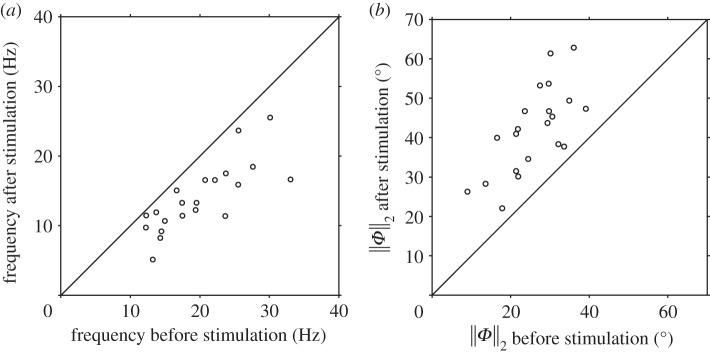
Cells exhibiting single-frequency beat after stimulation: distribution of the (*a*) beat frequency and (*b*) RMS proxidistal angle ∥*Φ*∥_2_. The diagonal lines have slopes of magnitude one, corresponding to zero-change in the parameters before and after stimulation.

**Figure 5.**
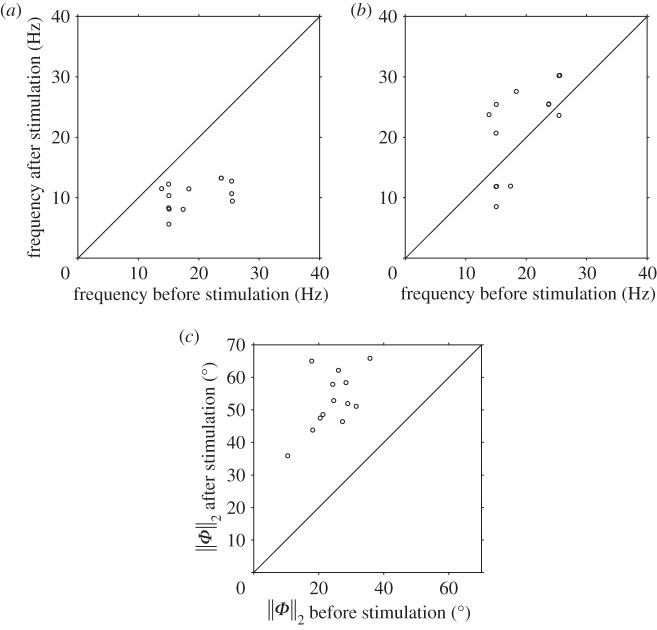
Cells exhibiting multiple-frequency beat after stimulation: distribution of the (*a*) mode I and (*b*) mode II beat frequency, and (*c*) RMS proxidistal angle ∥*Φ*∥_2_ after stimulation against their respective values before stimulation.

Data on the lower (‘mode I’) frequencies are summarized in table 1. The beat frequency of cells prior to stimulation had median 18.9 Hz and interquartile range 15.0–24.2 Hz (*n*=34). Beat frequency was reduced in every cell following stimulation (figures 4*a* and 5*a*), and in 32 of 34 cells by a larger amount than any cell in the dummy perfusion group. The single- and multiple-frequency subpopulations did not differ significantly prior to stimulation. The response of single-frequency cells to 4AP stimulation was similarly variable, with median change in frequency of −29% and interquartile range −37 to −20%; however, in all cases the frequency was reduced (figure 4*a*; *p*<0.001). In cells with multiple frequency peaks, the mode I following stimulation was always lower than the pre-stimulation frequency (figure 5*a*; median change −45%, interquartile range −54 to −38%, *p*<0.001), whereas the next largest (‘mode II’) frequency showed no clear pattern (figure 5*b*; median change +8%, interquartile range −21 to +38% change, *p*=0.27). Examining all cells and taking the mode I frequency for the multiple-frequency responders, the median percentage change following stimulation was −36%, with interquartile range −46 to −21%, again a highly significant change (*p*<0.001). The single- and multiple-frequency subpopulations differed significantly in mode I frequencies post-stimulation (*p*=0.022).

**Table 1.** Median frequencies before and after stimulation, and percentage changes (interquartile ranges given in parentheses) for four donors and *n*=34 cells. (In the first two rows, data are given for the subpopulations exhibiting single- (*n*= 21) or multiple-frequency (*n*=13) spectra post-stimulation. The mode I value post-stimulation only is given for multiple-frequency cells. Symbols: asterisk (*, **, ***) indicate significant differences pre- versus post-stimulation at the 5%, 1% and 0.1% levels, respectively; and dagger (^†^, ^††^ and ^†††^) indicate corresponding significance levels in the differences in response between single- and multiple-frequency subpopulations.)

	before 4AP	after 4AP (mode I)	
	frequency (Hz)	frequency (Hz)	percentage change
single frequency	19.4 (14.4, 23.7)	13.3 (11.4, 16.5)	−29 (−37, −20)***,^†^
multiple frequency	17.4 (15.0, 23.7)	10.7 (8.3, 12.2)	−45 (−54, −38)***,^†^
all cells	17.9 (15.0, 23.7)	11.7 (9.9, 14.6)	−36 (−46,−21)***

### 4.4 Proxidistal angle increases following 4-aminopyridine stimulus, and by a greater degree in multiple-frequency responders

Figures 4*b* and 5*c* show the RMS proxidistal angle data for single and multiple-frequency cells, respectively, with summary statistics and same-cell percentage changes given in table 2. In each group (single frequency, multiple frequency and all cells), the increase in ∥*Φ*∥_2_ was highly significant (*p*<0.001). Proxidistal angle post-stimulation was also significantly greater in the multiple-frequency responding cells than in the single-frequency cells (*p*=0.002), with median changes in ∥*Φ*∥_2_ of +57% in the single-frequency subpopulation and +130% in the multiple-frequency subpopulation.

**Table 2.** Median values of RMS proxidistal angle ∥*Φ*∥_2_ before and after stimulation, and same-cell percentage changes (interquartile ranges given in parentheses). (In the first two rows, data are given for the subpopulations exhibiting single- or multiple-frequency beat characteristics post-stimulation. For details on the symbols (*, ^†^), see table 1.)

	RMS proxidistal angle (°)		
	before 4AP	after 4AP	percentage change
single frequency	27.5 (21.4, 30.6)	42.2 (34.6, 47.3)	+57 (+41, +94)***,^††^
multiple frequency	24.6 (20.5, 28.3)	51.9 (47.5, 58.4)	+130 (+84, +140)***,^††^
all cells	25.4 (21.3, 30.0)	46.7 (38.7, 53.2)	+88 (+48, +120)***

### 4.5 Power dissipation is reduced following 4-aminopyridine stimulus, and by a greater degree in multiple-frequency responders

**Figure 6.**
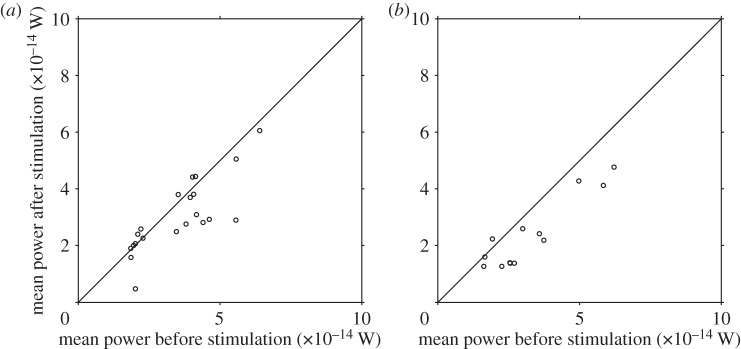
Distribution of mean hydrodynamic power dissipation in (*a*) single-frequency cells, and (*b*) multiple-frequency cells.

Figure 6 shows the change in power dissipation, with summary statistics and same-cell percentage change data given in table 3. The single-frequency subpopulation exhibited a median change of −7% (interquartile range −27 to +3%), which almost reached statistical significance at the 5% level (*p*=0.050). The multiple-frequency cells by contrast showed a larger reduction (median −29%, interquartile range −44 to −14%) which was highly significant (*p*<0.001). Across the whole sample, the median change in power dissipation was −15% (interquartile range −35 to +1%), again a highly statistically significant reduction (*p*<0.001). The differences in response between the single- and multiple-frequency groups was significant at the 5% level (*p*=0.04).

**Table 3.** Median power dissipation before and after stimulation, and same-cell percentage changes (interquartile ranges given in parentheses). (In the first two rows, data are given for the subpopulations exhibiting single- or multiple-frequency beat characteristics post-stimulation. For details on the symbols (*, ^†^), see table 1. The change in power dissipation for the single-frequency subpopulation is also borderline statistically significant (*p*=0.050).)

	power (×10^−14^ W)		
	before 4AP	after 4AP	percentage change
single frequency	3.8 (2.1, 4.2)	2.8 (2.3, 3.8)	−7 (−27, +3)^†^
multiple frequency	2.7 (2.3, 3.7)	2.2 (1.4, 2.6)	−27 (−44, −14)***,^†^
all cells	3.5 (2.1, 4.2)	2.5 (1.9, 3.8)	−15 (−35, +1)***

## 5. Discussion

The flagellar motion and hydrodynamic power dissipation of human sperm adhered to coated coverglass before and after hyperactivating stimulus with 4AP was quantified and reported. Beat frequency was reduced consistently following 4AP stimulation across the cells studied, although the degree of reduction was heterogeneous; moreover, around one-third of cells exhibited multiple-frequency beat behaviour following stimulation. By contrast, this behaviour occurred in only one of the 10 cells exposed to dummy perfusion; this latter observation may be a consequence of spontaneous hyperactivation, possibly owing to mechanotransduction of perfusion flow stress. Two of the 36 cells could not be analysed via the imaging methodology owing to non-planarity of the flagellar beat.

The post-stimulation beat frequency we observed (median 11.7 Hz) is comparable to the incubation-induced free-swimming average of 14.6 Hz reported in human cells [8]. Taking into account the fact that Morales *et al.* [28] and Mortimer *et al.* [10] used a measure of frequency equivalent to double the measure used in this paper, their reported values of 19.1–21.5 Hz and 22 Hz are also comparable to our observations. Comparing our observations with related work on other mammalian species, we note that monkey sperm exhibit a much lower frequency under hyperactivating conditions (4.4 and 3.8 Hz for symmetric and asymmetric cells [29]), as do hamster sperm (6.7 Hz [30]) and shrew sperm (1.9 Hz [17]).

A feature of working with adhered cells is that gross measures of cell motility, such as amplitude of lateral head movement or curvilinear velocity, can no longer be used and it is therefore essential to analyse the beating of the flagellum. The ‘proxidistal angle’ *Φ* was defined as a quantitative measure to reflect the increased bending, particularly in and near the midpiece, that occurs in the hyperactivated state. This quantity proved to be a consistent marker of 4AP stimulation, increasing in every cell following stimulation; we believe this measure may also provide a useful and visually interpretable quantitative measure of degree of activation in free-swimming cells, for example, in a computer-aided semen analysis setting. In the longer term, novel techniques such as lensfree optofluidic microscopy, which image populations of cells in three dimensions [31], may be combined with flagellar capture, enabling measures such as proxidistal angle to be applied in free-swimming populations unconstrained by surfaces.

In the one-third of cells exhibiting multiple-frequency beating under 4AP stimulation, the lower frequency mode was consistently lower than the pre-stimulation frequency. Proxidistal angle increased to a greater degree in these cells than the single-frequency subpopulation, indicating a greater degree in the change to flagellar bending.

The quantity $\bar{P}$ we have calculated is the average rate of energy dissipation by viscosity; as all mechanical work done on the fluid is through the movement of the flagellum, $\bar{P}$ is a lower bound for the rate of ATP production by the cell. The greater apparent vigour of hyperactivated beating has previously been interpreted as indicating greater energetic requirements [32]. However, single-frequency responders showed a borderline statistically significant decrease in power dissipation (*p*=0.050), and multiple-frequency cells decreased their power dissipation in almost all cases. Moreover, the reduction in power was significantly greater in the multiple-frequency group compared with the single frequency cells (*p*=0.04). This significant difference in a mechanical quantity suggests that single- and multiple-frequency beating may reflect an underlying difference in cell function, with multiple-frequency beating representing a more emphatic, though not more energetic, response.

Sperm bioenergetics is a topic of significant interest due to the potential implications for fertility of impairment of metabolism, for example, Odet *et al.* [33] showed how lactate dehydrogenase C is involved in both glycolysis and ATP homeostasis, and its disruption leads to loss of progressive and hyperactivated motility and male infertility in mice; Piomboni *et al.* [34] recently reviewed data on mitochondrial defects associated with asthenozoospermia and argued for further investigation into mitochondrial energy metabolism owing to its relevance to physiological and pathological function. Viscous power dissipation *P*, which quantifies the rate at which work is done on the fluid by the flagellum is only part of the total energy budget of the cell; other factors include any dissipative structures within the flagellum, and the metabolic requirements of other processes, for example, ion pumps. Moreover, we did not attempt to localize energy dissipation within the flagellum. However, the energy requirements of sperm motility are a subject of interest and discussion in biochemical approaches to andrology [33–36] and so we argue that an important adjunct to these studies is to include some quantification of viscous dissipation, and how this quantity changes with the different waveforms associated with different types of motility.

An early study of guinea pig and hamster sperm capacitation [18] suggested that power did not significantly change in these species. By contrast, data on rabbit sperm recovered by isthmic flushing [19] showed a 20-fold increase in power dissipation, although this change was relative to the subdued state of cells immediately after recovery. Dissipation associated with flagellar movement scales with the product of: (i) the force per unit length exerted by the flagellum on the fluid due to viscous drag, (ii) the length of the flagellum, and (iii) the velocity of the flagellum as it exerts this force. Furthermore, the force per unit length exerted by the flagellum on the fluid is proportional to the product of: (i) the velocity of the flagellum, and (ii) the viscosity of the fluid. Taking velocity as proportional to length multiplied by frequency, Dresdner & Katz [20] then proposed the theoretical scaling *P*∼*μf*^2^*L*^3^, where *f* is frequency, immediately providing insight into how a modest frequency reduction can result in a significant reduction in power dissipation. If we instead take into account the fact that velocity will more precisely be proportional to (beat amplitude) × (force) rather than length *per se*, an alternative scaling is *P*∼*μf*^2^*A*^2^*L*, where *A* is amplitude. The effects of hyperactivation on power dissipation may therefore depend on the trade-off between decrease in frequency and increase in beat amplitude. The effect of activation state, fluid medium rheology and motility-modulating drugs, on power dissipation warrant further attention, with interspecies variability in metabolism being an important factor to take into account [35,36].

Williams & Ford [32] found that glycolytic ATP production is required for vigorous motility and hyperactivation of human sperm cells over an extended 18 h incubation. Ho *et al.* [37], working with demembranated bull sperm, found that the ability of Ca^2+^ to induce hyperactivation was dependent upon ATP availability. Taken together, these findings appear to suggest that the flagellar bending associated with hyperactivated motility requires a greater amount of energy than non-hyperactivated motility, and indeed both groups cited made this interpretation. We believe that our findings that hyperactivated motility need not involve greater energy dissipation warrant a more refined interpretation; nevertheless, we acknowledge that this study considers only viscous dissipation and not the instantaneous energetic requirements of non-dissipative storage of bending energy in the elastic structures of the flagellum. This aspect will be addressed in future work.

While sperm encounter a 37°C environment in physiology—and indeed are speculated to respond to temperature gradients [38] *in vivo*—we instead performed experiments at room temperature in order to achieve a more consistent temperature with perfusion. Motility, and particularly the ability to hyperactivate, are temperature-sensitive [39], so investigation of the effect of physiological temperature should constitute part of future studies involving environmental conditions closer to those found in the female reproductive tract; future investigation is also required into the effect of non-Newtonian media, an additional important feature of the *in situ* environment that profoundly affects the hyperactivation response [40].

An advantage of the use of tethering and perfusion is the ability to examine changes in individual cells and associated applicability of pairwise statistical testing. Tethering is also observed in experiments with female reproductive tract [41] and is clearly relevant to the final stages of binding and penetration of the egg. Moreover, analysis of adhered cells is relevant to recent developments in assisted reproduction, for example, the physiological intracytoplasmic sperm injection (PICSI) technique [42], which identifies cells capable of binding to hyaluronic acid as a proxy for cumulus-binding.

Asymmetric hyperactivated waves may produce ‘tugging’ forces which assist with detachment from fallopian tube epithelium [15]; a study of monkey sperm revealed a significant increase in transverse forces under hyperactivation [14], inferred to be important in zona penetration. Recent experiments in the mouse [43] have revealed that hyperactivated beating is associated with ‘bind-and-detach’ motility in the tract, a finding which can be interpreted through mathematical modelling [16]. To test these models, a future application of the technique we have developed would be directly to analyse forces produced by sperm interacting with *ex vivo* tract; this work is technically challenging in a co-culture setting.

Two of the four donors were proven fertile, the remaining two presented sufficiently high-quality samples to qualify as treatment donors. Burkman [8] showed that sperm from oligozoospermic patients undergo incubation-induced hyperactivation at a much lower rate than sperm from fertile donors; it is possible that lower rates of responsiveness to 4AP could be indicative of an underlying pathology.

Despite decades of research, there are no robust pharmacological treatments for sperm motility deficiency, one of the major causes of subfertility. The larger system of motility modulation, of which hyperactivation is a part, is being unravelled by integrated experiments and models—for recent developments and review see [16,44–48]. The deeper understanding these approaches will provide will be crucial to developing urgently needed novel treatments. The method employed in this study may prove useful in assessing the effects of putative motility-stimulating drugs, interpreting the effects of physiological ligands such as progesterone, in addition to pharmacological toxicology assessments. The combination of novel approaches from physical and computational sciences with cell biology and pharmacology provides the best approach to understanding this vital and challenging natural system.

## Data accessibility

Supporting data have been submitted to the University of Birmingham institutional repository epapers.bham.ac.uk/1921.
